# Books and Authors

**Published:** 1907-06

**Authors:** 


### BOOKS AND AUTHORS.

**The Piactice of Obstetrics.** By Eminent Authorities. Edited by **Reuben Peterson,** A.B., M.D., Professor of Obstetrics and Diseases of Women in the University of Michigan, Ann Arbor, Octavo, 1087 pages, with 523 engavings and 30 full-page plates in colors and monochrome. Lea Brothers & Co., Philadelphia and New York, 1907. (Cloth, $6.00; leather, $7.00; half morocco, $8.00, net prices).

The practitioner's library in three volumes covering the fields of obstetrics, gynecology, and pediatrics, is a combination that appeals to the average physician as being at once practical and scientific,—practical because useful, scientific because correlated. The preceding volumes have considered gynecology and pediatrics, respectively, hence this one on obstetrics closes the series. The contributors,—for it will be remembered that the work is encyclopedia in character,—are Charles Sumner Baacon, Montgomery A. Crockett, W. A. Newman Dorland, G. Carl Huber, Hugo Ehrenfest, Henry Foster Lewis, Walter P. Manton, John F. Moran, Benjamin R. Schenck and Alfred Scott Warthin.

The first section, consisting of three chapters, treats of the physiology and development of the ovum, and is written by Dr. Huber. It is a scientific presentation of the subject from the latest embryologic data. The second section, consisting of five chapters, deals with the physiology of pregnancy, and is written by Dr. Manton, who is eminently qualified for the task assigned him. The chapter on differential diagnosis, a very important subject, is particularly to be commended. The third section, six chapters, presents the physiology of labor, and is written by Dr. Dorland. It is a practical dissertation, the chapter on the management of labor being especially deserving of praise. The physiology of the puerperium, section four, in three chapters, by Dr. Lewis, treats of an important branch in a satisfactory manner.

The pathology of pregnancy forms the subject of the fifth section, consisting of thirteen chapters, written by Drs. Ehrenfest, Warthin, Lewis and Schenck, deals with a topic of the utmost importance, and each of the contributors has met the duty assigned him with ability. The next section, number six, in eleven chapters, handles the pathology of labor, and is written by Drs. Moran and Ehrenfest. It is a scientific disquisition upon an essential part of obstetrics, and will prove instructive to physicians as well as students. The pathology of the puerperim forms the subject matter of section seven, which is made up of three chapters and is written by Drs. Lewis and Bacon.

Obstetric operations naturally form an attractive section, number eight, which is presented by Dr. Crockett, formerly of Buffalo, now of Pinehurst, N. C. Dr. Crockett's experience is such as to entitle him to be heard on this topic, and he deals with it admirably. The final section, number nine, in six chapters deals with the newborn infant, and is written by Drs. Lewis and Bacon.

The work viewed in its entirety is an exposition of the science and art of obstetrics in its present state, is a credit to all concerned in creating it, and will stand as an addition to the literature of medicine. It is profusely and artistically illustrated, many of the engravings being original and all are instructive.

---

**Conservative Gynecology and Electrotherapeutics.** By **G. Betton Massey,** M.D., Attending Surgeon to the American Oncologic Hospital, Philadelphia. Fifth edition. Illustrated. Octavo, pp. 467. Philadelphia: F. A. Davis Co. 1906.

It cannot be denied that this author, whether one differs from him or agrees with him, displays great energy in exploiting his belief as to the value of electricity in the treatment of diseases of women. He has pursued the subject through five editions of his book, the first having been issued in 1889, and he now asks the medical profession to accept this work as the authoritive exponent of "conservatism in gynecology," though he regards the specialty still in its "formative stages."

The author in this edition has considered the constant current derived from street mains in its latest phase, and has further elaborated the cataphoric treatment of cancer. Whoever may wish to obtain knowledge concerning the uses of electricity can gain it from a careful study of this book, the author being most painstaking in this direction. The general make-up of the book and the illustrations remain as in the last previous edition, it being beyond criticism in these respects.

---

**International Clinics.** A Quarterly of Illustrated Clinical Lectures and especially prepared articles on Treatment, Medicine, Surgery, Neurology, Pediatrics, Obstetrics, Gynecology, Orthopedics, Pathology, Dermatology, Ophthalmology, Otology, Rhinology, Laryngology, Hygiene and other topics of interest to students and practitioners. Edited by **W. T. Longcope,** M.D. Volume 1, Seventeenth series. 1907. Philadelphia and London: J. B. Lippincott Co. (Cloth, $2.00).

The first volume of the seventeenth series contains some excellent literature as will be observed by noting the following list of titles and authors. The first section relates to treatment, the first number being on the psychic treatment of some of the functional neuroses, by Lewellys F. Barker; the second is entitled, recent advances in the prevention and cure of tuberculosis, by Frederick P. Gay; the third is on the diagnosis and treatment of gastric ulcer, by David Somerville; and the fourth considers the treatment of functional heart disease, being a clinical lecture by James J. Walsh.

In the section on medicine we find the clinical diagnosis of enlargement of the thymus, by Alfred Scott Warthin; the functional capacity of the heart, by George William Norris; exhaustion and toxemia as underlying factors in the production of neurasthenia, hysteria, and delirium, by Theodore Diller.

Under the head of surgery the titles are, report of two months' service at Bellevue Hospital, by Frederick S. Dennis; a clinical lecture on neurotic affections of the joints, by Charles A. Morton; monarticular inflammation of the knee joint, (and several other subjects) by Nicholas Senn: on the opening of the pleural cavity without pneumothorax, by Th. Tuffier.

In obstetrics and gnyecology the contribution is, disorders of the umbilicus with special reference to the newborn and the infant; umbilical infections, by A. Ernest Gallant. In ophthalmology is an article on intraocular angiosclerosis and its prognostic and diagnostic significance, by George E. deSchweinitz. In laryngology there is also one article—hysterical mutism, by G. Hudson Maknen.

Under progress and medicine during 1906 treatment is reported by A. A. Stevens; medicine by David L. Edsall and Verner Nisbet; and surgery by Joseph C. Bloodgood.

The foregoing synopsis of the material which this volume contains indicates literature of great value and stamps this number as one of the best of the series.

---

**Biographic Clinics.** By **George M. Gould,** M.D., Editor of American Medicine. Vols. IV and V. Influence of Visual Function, Pathologic and Physiologic, upon the Health of Patients. 12mo, about 400 pages each. Philadelphia: P. Blakiston's Son & Co. (Price, $1.00 each, net).

For a considerable period of time the influence of the visual function upon health has been recognised, to a certain extent at least, but it has remained for Dr. Gould to accentuate, develop and give more exact place to much of the symptom complex bearing on the subject.

These two volumes of the biographic clinics series contain essays concerning the influence of visual function, pathologic and physiologic, upon the health of patients. They have been collected from journalistic and magazine sources and reflect the author's views on the subject with great emphasis. They furnish interesting and instructive reading and may be studied with profit by even those who do not endorse the author's views in toto.

In volume four are many medico-biographic sketches which show defective visual function, some of which are of unusual interest. Especially so is that of Lafcadio Hearn, who had but one eye and that evidently enormously near sighted; the other had been lost in youth, yet what beautiful pictures he wrote of Japanese life and character.

And so on unto the end are many entertaining and instructive sketches that serve to reveal the inner life of men of talent who were eccentric.

---

**Progressive Medicine,** Vol. I, March, 1907. A Quarterly Digest of Advances, Discoveries and Improvements in the Medical and Surgical Sciences. Edited by **Hobart Amory Hare,** M.D., Professor of Therapeutics and Materia Medica in the Jefferson Medical College of Philadelphia. Octavo, 280 pages, with illustrations. Lea Brothers & Co., Philadelphia and New York. (Per annum, in four cloth-bound volumes, $9.00; in paper binding, $6.00, carriage paid to any address).

The contributors to this number are William T. Belfield, Joseph C. Bloodgood, John Rose Bradford, John G. Clark, William B. Coley, Floyd M. Crandall, Edward P. Davis, William Ewart, Edward Milton Foote, Charles H. Frazier, William S. Gottheil, Edward Jackson, D. Braden Kyle, H. R. M. Landis, Robert B. Preble, B. Alexander Randall, William G. Spiller, J. Dutton Steele, and Alfred Stengel.

The topics dealt with in this number are surgery of the head, neck and thorax, by Charles H. Frazier; infectious diseases, including acute rheumatism and croupous pneumonia, by Robert B. Preble; the diseases of children, by Floyd M. Crandall; rhinology and laryngology, by D. Braden Kyle; otology, by B. Alexander Randall.

The book is full of material interesting to both physician and surgeon. The low subscription price, together wth its valuable contents, should place it on every progressive physician's table.

